# Evaluation of the effect of cannabidiol administration with and without nonsteroidal anti-inflammatory drugs in dogs with mobility disorders: a prospective, double-blind, crossover, placebo-controlled study

**DOI:** 10.3389/fvets.2024.1449343

**Published:** 2024-09-25

**Authors:** Bryce Talsma, Lindsay Hochman Elam, Stephanie McGrath, Tianjian Zhou, Craig B. Webb, Felix Michael Duerr

**Affiliations:** ^1^Department of Clinical Sciences, Colorado State University, Fort Collins, CO, United States; ^2^Department of Statistics, Colorado State University, Fort Collins, CO, United States

**Keywords:** cannabidiol, CBD, dog, mobility, NSAID, osteoarthritis

## Abstract

**Introduction:**

With rapidly growing interest in the use of cannabidiol (CBD) in the management of pain and other conditions, more information is needed on the safety and efficacy of this supplement, particularly its co-administration with commonly used pharmaceuticals such as non-steroidal anti-inflammatory drugs (NSAIDs). This study sought to assess the effect of CBD in dogs with mobility impairments, as well as evaluate the clinical tolerance of CBD used together with NSAIDs.

**Materials and methods:**

Forty-two client-owned dogs with diagnosed mobility impairments were enrolled in this prospective, double-blind, crossover, placebo-controlled study. Baseline data were collected for 10–14 days followed by random allocation to either placebo or CBD oil for 45 days with a 30-day washout period in between. CBD was dosed at 5 mg/kg orally every 12 h with masked placebo administered at equal volume. Outcome measures included objective gait analysis, accelerometry, and clinical metrology instruments. CBD plasma levels and serum biochemistry were also collected along with hepatic ultrasound if warranted.

**Results:**

Thirty-eight dogs finished the study with thirty-nine included for at least partial analysis. Compared to baseline, dogs receiving CBD showed evidence of improved outcomes based on blinded veterinary assessments and accelerometer data. Compared to placebo, dogs receiving CBD showed some evidence of improved outcomes on CBPI, CSOM, and blinded veterinary assessments, but not for objective outcome measures. There was evidence of increased ALP when CBD was co-administered with NSAIDs compared to CBD administration alone. Additionally, there was evidence of ALT elevations with CBD and NSAID co-administration, but this elevation did not show evidence of an increase over CBD use alone.

**Discussion:**

These results suggest a potential therapeutic benefit in the administration of CBD for the management of mobility impairments, but greater ALP elevations were seen when administered with NSAIDs. While the sample size of dogs that received further hepatic work-up for liver enzyme elevations is small, chosen diagnostics varied, and liver biopsies were not performed, there did not appear to be clinically apparent liver damage. Further research is needed to better understand the efficacy of CBD in a larger population of dogs and patient tolerance and safety when administered with NSAIDs or other medications long term.

## 1 Introduction

Mobility is a key component to perceived quality of life in both human and veterinary patients (1). In canines, a large population study reported musculoskeletal disease and the inability to stand as the leading cause of euthanasia in German Shepherd dogs, surpassing neoplasia (2). Mobility impairments are commonly treated with a multimodal approach to manage a patient’s clinical signs with nonsteroidal anti-inflammatory drugs (NSAIDs) currently considered the first-line standard of care. Despite a systematic review indicating low instances of severe adverse events related to NSAID administration (3), there is concern for its long-term use in patients among both veterinarians and owners (4). Monoclonal antibody medication targeted for osteoarthritis (OA) pain appears to offer another promising option for patients, but current research has not evaluated its administration long-term or in combination with NSAIDs (40). Other currently available pain medications, while generally well tolerated, appear less effective in the management of pain (5, 6). This underlines the necessity for alternative analgesic agents that are safe, efficacious, and easy to administer.

The therapeutic use of cannabinoids is of recent interest to both human and veterinary medicine (7, 8). While literature supporting its use remains limited (9), the recent declassification of industrial hemp has improved access for research in veterinary medicine. Current evidence suggests that the use of cannabidiol in dogs for the management of OA is promising, but further investigation is needed to determine the efficacy, dose, formulation, and safety of combinations with other medications (10–12). Most available studies lack the use of objective data, such as kinetic analysis and accelerometry, to evaluate efficacy. To the authors’ knowledge, only one study has evaluated the efficacy of cannabidiol and NSAIDs together in dogs affected with OA using objective outcome measures (13). Given that NSAIDs remain the mainstay of therapeutic management of OA, it is desirable to find additional therapies that are both safe and effective with co-administration.

This prospective, double-blind, crossover, placebo-controlled study sought to assess the effect of CBD in dogs with mobility impairments, as well as evaluate the clinical tolerance of CBD used together with NSAIDs.

## 2 Materials and methods

The study protocol was approved by the Clinical Review Board of Colorado State University (IACUC: #1608, initial approval 4/1/2021), and owner consent was obtained prior to enrollment. Client-owned dogs of any breed or sex presenting to Colorado State University Veterinary Teaching Hospital with lameness or mobility impairments that resulted in measurable pain were eligible for participation. Included dogs must have been ≥10 kg, be in general good health (defined as being able to perform everyday activities such as independent eating/drinking, walking, and independently rising and laying down), not be on an active weight loss plan, adapted to wearing a collar at all times, and have a Canine Brief Pain Inventory (CBPI) average pain severity score (PSS) and pain interference score (PIS) ≥ 2 for each. If the dog had a change in the average PSS and/or PIS between baseline visits 10–14 days apart, disease was considered not stable at the time, and the dog was excluded from the study or re-evaluated when disease was considered to plateau. It was also required that the dogs were on a consistent management plan for at least 4 weeks prior to enrollment. This could include NSAIDs and other medications and supplements if they were administered consistently prior to enrollment and continued throughout the study. Dogs receiving grapiprant were excluded from enrollment, as the study sought to look at the effect of traditional NSAIDs in combination with CBD. Other exclusion criteria included disease expected to substantially change throughout the study period (i.e., neoplasia, partial rupture of the cranial cruciate ligament, degenerative myelopathy, etc.), surgery or joint injections within 3 months of enrollment, or administration of corticosteroids within the last month. Only dogs with chronic, stable stifles with osteoarthritis were enrolled to reduce the possibility of clinical worsening due to progressive tearing of the CCL or development of a meniscal tear. Dog with evidence of pre-existing liver or kidney disease (any elevation of ALT, AST, GGT, T-bilirubin, bile acids, or BUN/creatinine, respectively) were also excluded. Mild elevations in ALP, defined as 2-6x above the high end of the reference range, were included given the low specificity (51%) of ALP as a marker for hepatobiliary disease (14).

At the time of enrollment, each participant received a complete orthopedic examination, objective gait analysis, baseline bloodwork profile (complete blood count and serum biochemistry), measurement of fasted bile acids, and baseline plasma CBD value. Dogs were required to have their mobility impairment diagnosed via an objective imaging modality that supported clinical exam findings prior to trial enrollment. Additional diagnostics including radiographs, musculoskeletal ultrasound, and/or neurologic exam by a board-certified veterinary neurologist (SM) were performed at the evaluating clinician’s discretion based on the patient’s prior diagnostics and clinical examination. While many dogs were diagnosed with bilateral disease (e.g., elbow osteoarthritis), the most clinically affected joint was used for enrollment and evaluation of outcome measures. The owners were informed that the use of new medications, supplements, dose changes, or new treatment strategies should be avoided throughout the trial, would need to be reported, and may result in exclusion from the study if it was considered to substantially impact the patient’s mobility. Minor deviations from the protocol such as a single rescue dose of an NSAID or new medications for a condition not affecting mobility (e.g., antibiotics) were deemed acceptable.

### 2.1 Treatment groups

**Figure 1:**
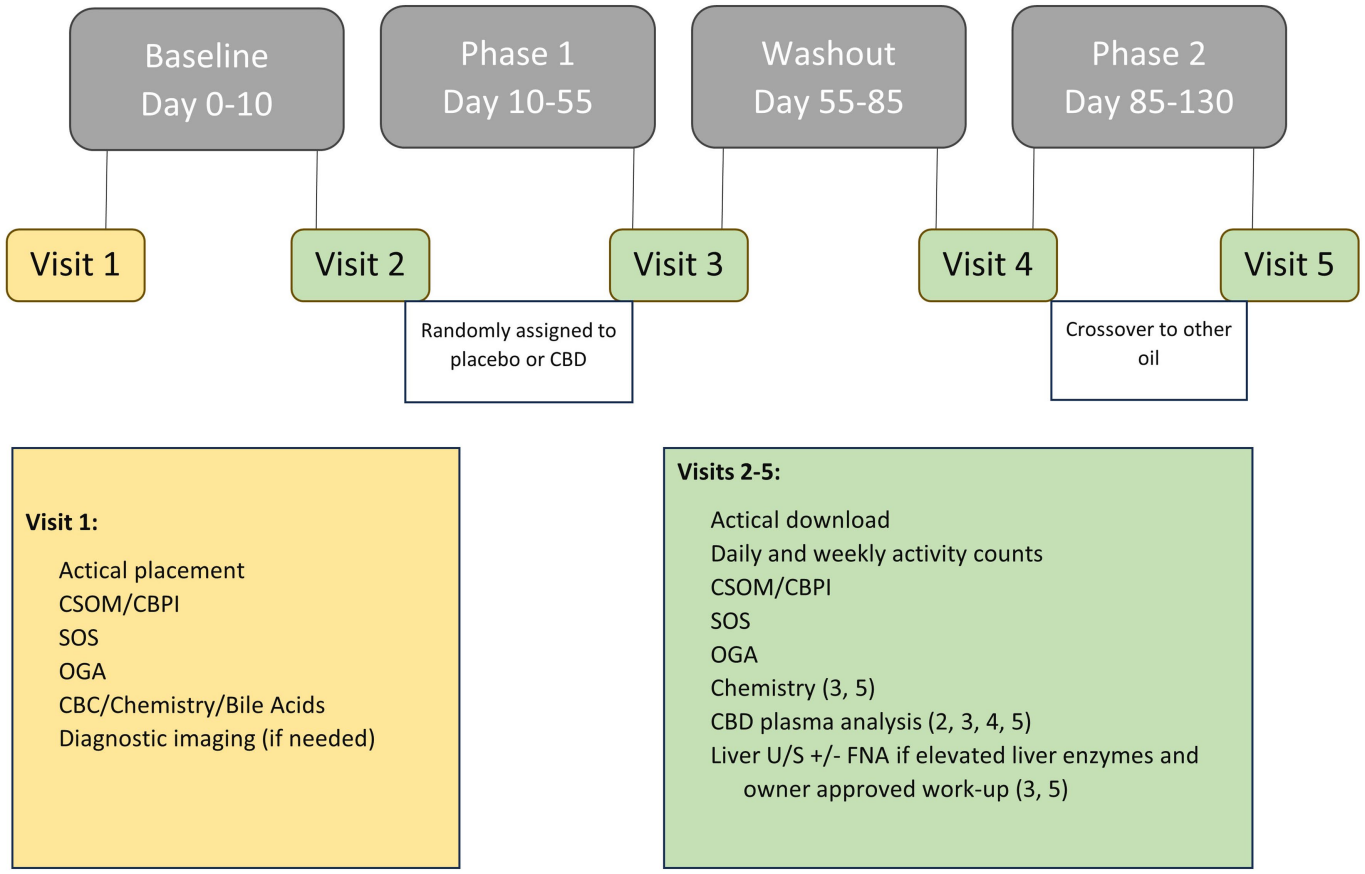
Overview of study timeline including data obtained at each visit, outcome measures, and any treatments administered at each phase.

Dogs were categorized as either receiving NSAID therapy or not receiving NSAID therapy during enrollment to ensure each group had the same number of dogs. Participants were allocated into one of two treatment groups using the random generator function in Microsoft Excel (Microsoft Corporation, Redmond, Washington): placebo followed by CBD treatment (PL-CBD) or CBD treatment followed by placebo (CBD-PL). After a 10–14 day baseline period, either placebo or CBD treatment was administered for 45 days depending on the patient’s group allocation. Following the first phase of treatment, dogs underwent a 30-day washout period prior to receiving the opposite treatment for a subsequent 45 days (Figure 1). If the 45th day fell on a weekend or holiday, or the owner was unable to make the appointment day, treatment was continued until the morning of evaluation.

The study sponsor (cbdMD, Charlotte, NC, United States) provided the CBD and placebo oil in two identical bottles to the research team. The bottles were coded by an unblinded individual who did not participate in veterinary assessments (FD). The oil, containing a medium chain triglyceride (MCT) oil and peanut flavoring for scent masking and palatability, was packaged in light protected bottles. The CBD oil contained approximately 1,500 mg CBD per 30 mL bottle, confirmed via third party testing (SC Laboratories California LLC, Santa Cruz, CA). Quality assurance testing was performed on the batch and a certificate of analysis was provided by the company (Supplementary material). The concentration of CBD was within 5% and above the labeled dose which is within the margins of error in analytical laboratories. CBD oil was dosed at 5 mg/kg CBD *per os* every 12 h, and the placebo was dosed at equal volumes and time intervals. The CBD or placebo oil was dispensed to the owners in individual bottles with a syringe marked at the appropriate volume for administration. The bottles contained identical labels and instructions for dosing each patient. The owners and all personnel involved in patient evaluation were blinded to the contents of the bottle. Owners were instructed to administer treatments with a meal.

### 2.2 Clinical pathology

Whole blood was collected at follow-up visits #3–5 for biochemistry and plasma CBD analysis (Figure 1). Elevations in liver enzymes including ALP, ALT, AST, T-bilirubin, and GGT were recorded and classified as mild (greater than two-fold but less than six-fold) or moderate (greater than six-fold) (14). Additionally, the percent increase from baseline was calculated.

### 2.3 Diagnostic hepatic ultrasound

If any liver enzyme elevations were noted, fasted bile acids in addition to a focused liver ultrasound and fine-needle aspirate were offered but not required. Hepatic ultrasounds were performed by a boarded radiologist or radiology resident under the direct supervision of a boarded radiologist. If changes were noted within the liver and owners consented, aspirates were collected and submitted for cytology. If necessary, dogs received 0.1 mg/kg butorphanol IV for hepatic ultrasound and aspirates. Findings on liver ultrasound and cytology were also documented.

### 2.4 Outcome measures

#### 2.4.1 Clinical metrology instruments (CMIs)

CMIs including the canine brief pain inventory (CBPI) and client-specific outcome measures (CSOM) were completed by the owners at each visit (Supplementary material). Initial CMIs were discussed with the owner at the time of enrollment to ensure understanding of the questionnaires, and the same owner filled out the CMIs at follow-up visits via dependent interviewing (15). For the CSOM, owners were provided a list of examples for both the activity and behavior portions of the form. Specific examples pertaining to the pet were also discussed, but ultimately, the owner was allowed to choose each activity and behavior. Owners were requested to select five activities and three behaviors pertaining to their dog’s mobility. For this reason, numeric indication of improvement could vary based on owner report of positive or negative behaviors. Values were adjusted for statistical analysis such that a higher numeric value indicated improvement for all patients regardless of reported behavior.

#### 2.4.2 Veterinary assessments

Patients were evaluated by a veterinarian at each visit, and a previously published orthopedic scoring system was used to quantify exam findings (16). The subjective orthopedic scoring (SOS) consisted of six components each rated with a score 0–4 (0 = normal, 4 = severe impairment) and evaluated lameness at a walk and trot, pain on manipulation, offloading of the most affected limb, willingness to load the contralateral limb, and functional disability. The sum of the scoring for each category was used for analysis.

#### 2.4.3 Accelerometry

All dogs enrolled in the study were fitted with an activity monitoring collar using the Actical (Respironics Mini Mitter Division, Bend, OR) collar as previously described (13). Monitoring was continuous throughout the study period with the epoch length set to 60 s. Data was downloaded at each visit to ensure activity was being recorded and battery life was sufficient. If there was damage to the device or errors in the download process, it was attempted to recover the data and a new Actical collar was placed on the dog. Otherwise, the same device was maintained for each pet throughout the study period.

#### 2.4.4 Objective gait analysis

Gait analysis was performed at each visit using a pressure sensitive walkway (PSW) (6-Tile High Resolution Strideway System, Tekscan Inc., South Boston, MA). Dogs were evaluated at a trot in a similar fashion to a previously described protocol (13). If the dog was unable to trot, they were evaluated at a walk. Prior to data collection, dogs were acclimated to the gait analysis laboratory and leash walking with the handler on the right and left. Six trials (three in each direction) with a subjectively constant velocity, in a straight line, without lateralization of the head, pulling on the leash, or stepping off the PSW were acquired. When only a single direction was tolerated, the dog was walked in that direction for six valid trials. Trials at subsequent visits were only considered valid for the individual patient if they fell within 0.3 m/s of the velocity established at the baseline visit. The correct labeling of foot placement was confirmed by video analysis collected during gait acquisition. Percent body weight distribution (%BWD) was calculated and averaged for the six valid trials at each visit.


$$\%BWD=\frac{PVF\;\left[N\right]\;\mathrm{of}\;\mathrm{the}\;\mathrm{limb}}{\mathrm{total}\;PVF\;\left[N\right]\mathrm{of}\;\mathrm{all}\;\mathrm{four}\;\mathrm{limbs}\;\mathrm{in}\;\mathrm{one}\;\mathrm{gait}\;\mathrm{cycle}}x\;100$$

### 2.5 Statistical analysis

Sample size calculation was performed using SAS Proc Power (SAS 9.4, SAS Institute Inc., Cary, NC). The baseline data from a previous study (*n* = 23 dogs with mobility impairment due to arthritis) was used for a paired *t*-test (corresponding to the crossover design) with alpha = 0.05. Power calculation was performed based on CBPI PSS and PIS. For CBPI PSS, the power calculation was based on a meaningful difference of 1 with a conjectured standard deviation of 1.68. To achieve 80% power *n* = 25 dogs are required; to achieve 90% power *n* = 32 dogs are required. For CBPI PIS, the power calculation was based on a meaningful difference of 2 with a conjectured standard deviation of 2.10 (17). To achieve 90% power and account for attrition, a sample size of *n* = 40 was proposed.

The outcome measures, including CMIs, OGA, accelerometry, and liver enzymes, were analyzed using a linear mixed model (18). The model was fit separately for each response variable using the lme4 package within the R statistical software (R 4.0.2, R Core Team, Vienna, Austria) (19). Each individual dog was included as a random effect to account for repeated measures. Treatment (baseline, post-CBD, and post-placebo), period (pre- and post-washout), and period-by-treatment interaction were included as fixed effects to identify the changes in outcome measures from baseline to post-treatment (CBD or placebo), as well as potential period and carryover effects due to the crossover study design (18). For liver enzyme data, NSAID administration and its interaction with treatment were also included in the model as fixed effects. Estimated marginal means and contrasts were calculated using the emmeans package (41). The *p*-values associated with the treatment effects were calculated based on *t*-tests of the regression coefficients in the linear mixed model and were used to determine statistical significance. Following the recommendations of experts in medical statistics, no multiplicity adjustments were performed given the exploratory nature of the analyses (20). The *p*-values should be interpreted for descriptive purposes but not for confirmatory decision making. Residual diagnostic plots were used to evaluate model assumptions (normality and equal variance of random errors). A log transformation was deemed necessary for the activity counts and for the following liver enzyme measures: ALP, ALT, and AST. After necessary transformations, no obvious violations of modeling assumptions were identified, as seen from the evenly scattered residuals around the horizontal zero line.

A Mann–Whitney U test was used to compare CBD plasma levels to placebo levels since the data was not normally distributed.

## 3 Results

**Figure 2:**
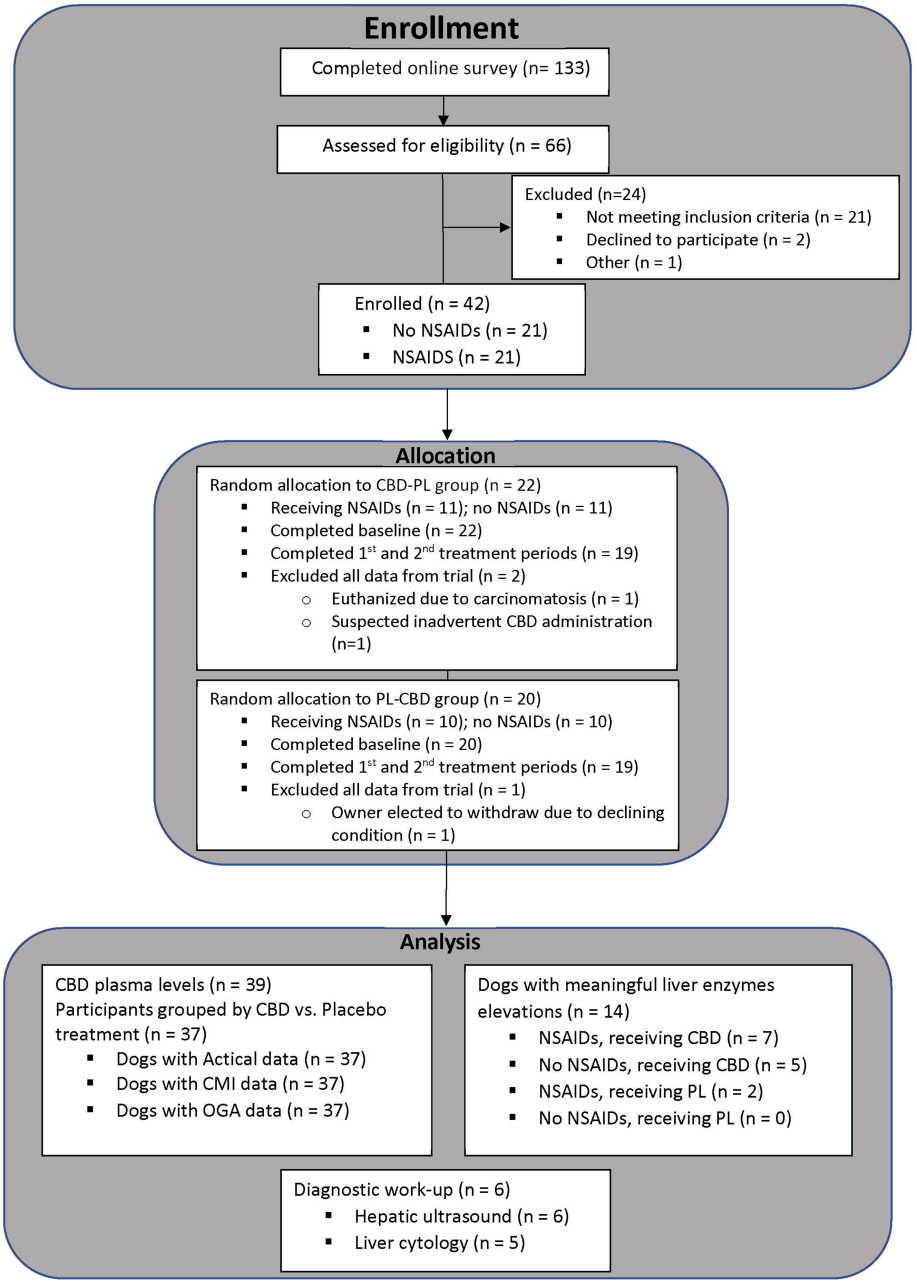
Number of participants at each phase of the clinical trial from the time of enrollment untill data analysis.

The number of surveys received, dogs evaluated, enrolled, and included for analysis is summarized in Figure 2. Forty-two dogs qualified for enrollment in the study. There were 21 each of neutered males and spayed females. Patient age ranged from 1 year to 15 years (media*n* = 7.5 years), and weight ranged from 15 kg to 68 kg (media*n* = 29 kg). Included breeds and the most clinically affected region are summarized in Table 1. Thirty-eight dogs completed the study. Two dogs were unenrolled after the owner elected withdrawal, one dog was euthanized for reasons unrelated to the study, and one dog was in a dog fight that resulted in a new lameness and exclusion from the remainder of the study. Two dogs who did not complete the study had data included until the time of withdrawal from the study. One dog who completed the study had all data removed after CBD plasma levels suggested inadvertent CBD administration during the placebo phase. In total, 39 dogs were included for at least partial analysis. Fourteen dogs had at least partial exclusion of data for which reasons are summarized in Table 1.

**Table 1:** Enrollment data including patient age, sex, breed, NSAID status, primary mobility disorder, sequence of treatment, and any reason for data exclusion.

Patient	Age	Sex	Breed	NSAID status	Diagnosis of most affected region	Sequence of treatment	Data exclusion
1	2	MN	Golden retriever	Carprofen	R elbow OA	PL-CBD	--
2	5	FS	Mixed breed	No NSAID	L stifle OA	PL-CBD	--
3	9	MN	Brittany spaniel	No NSAID	R gluteal tendinopathy	PL-CBD	--
4	9	MN	Border collie	No NSAID	R biceps tendinopathy	CBD-PL	Developed CCT injury, excluded visit #3–5
5	2	MN	Mixed breed	No NSAID	R hip OA	CBD-PL	--
6	15	FS	Norwegian elkhound	No NSAID	T3-L3 myelopathy	PL-CBD	Excluded all data, owner withdrawal after visit #3 due to declining condition
7	2	MN	Mixed breed	No NSAID	R carpal OA	PL-CBD	--
8	5	FS	Labrador retriever	No NSAID	L elbow OA	CBD-PL	--
9	2	FS	Mixed breed	No NSAID	R stifle OA	PL-CBD	--
10	12	FS	Labrador retriever	Carprofen	L hip OA	PL-CBD	--
11	7	FS	English bulldog	No NSAID	L elbow OA	CBD-PL	Data excluded at visits #4–5 due to new lameness
12	11	FS	Mixed breed	No NSAID	R biceps tendinopathy	PL-CBD	--
13	13	FS	Labrador retriever	Carprofen	L hip OA	CBD-PL	--
14	5	FS	American staffordshire terrier	Carprofen	R carpal OA	PL-CBD	Data excluded at visit #3 due to development of pododermatitis
15	2	FS	Belgian malinois	No NSAID	R hip OA	PL-CBD	--
16	9	FS	Labrador retriever	No NSAID	L shoulder OA	CBD-PL	--
17	7	FS	Mixed breed	No NSAID	LS disease	CBD-PL	Unenrolled from the study at visit #4 due to dog attack resulting in new lameness
18	11	MN	Mixed breed	Carprofen	L hip OA	CBD-PL	--
19	13	FS	Golden doodle	No NSAID	L hip OA	CBD-PL	Excluded all data, diagnosed with carcinomatosis during study and euthanized
20	5	FS	Mixed breed	Carprofen	L carpal OA	CBD-PL	Plasma CBD levels excluded due to low levels throughout study
21	10	MN	Labrador retriever	No NSAID	L shoulder OA	CBD-PL	Data excluded at visit #4–5 due to R shoulder injury while hunting
22	8	FS	Golden retriever	No NSAID	L proximal gastrocnemius tendinopathy	CBD-PL	--
23	2	MN	Mixed breed	Carprofen	R hip OA	CBD-PL	--
24	10	FS	Mixed breed	No NSAID	L carpal flexor tendinopathy	CBD-PL	Data excluded at visit #4–5 due to dog attack resulting in shoulder injury
25	10	MN	Mixed breed	No NSAID	LS disease	PL-CBD	Data excluded at visit #4–5 due to starting prednisone for pemphigus foliaceous
26	7	MN	Mixed breed	No NSAID	L biceps tendinopathy	CBD-PL	--
27	9	FS	Mixed breed	Carprofen	L biceps tendinopathy	CBD-PL	Data excluded at visit #3–5 due to R shoulder injury
28	10	MN	Mixed breed	Carprofen	R elbow OA	CBD-PL	--
29	5	MN	Bernese mountain dog	Carprofen	R elbow OA	PL-CBD	Data excluded at visit #3 due to deviation from protocol – stopped NSAID administration for 1 week
30	9	MN	Mixed breed	Carprofen	R hip OA	PL-CBD	Excluded visit #3–5, developed HGE and hospitalized
31	1	MN	Mixed breed	No NSAID	R elbow OA	PL-CBD	--
32	6	MN	Mixed breed	Carprofen	R elbow OA	CBD-PL	--
33	14	FS	Golden retriever	Carprofen	L stifle OA	CBD-PL	Excluded visit #5 due to R full CCL tear. CBD Plasma data excluded for low CBD levels at baseline
34	3	MN	Labrador retriever	Carprofen	L stifle OA	PL-CBD	--
35	11	FS	Mixed breed	Carprofen	R stifle OA	PL-CBD	--
36	10	MN	Border collie	None	L hip OA	PL-CBD	--
37	2	MN	American staffordshire terrier	Carprofen	L tarsal OA	PL-CBD	--
38	10	MN	Labrador retriever	Carprofen	R shoulder OA	PL-CBD	--
39	2	FS	German shepherd dog	Carprofen	R elbow OA	PL-CBD	--
40	9	MN	Australian shepherd	Carprofen	LS disease	CBD-PL	Excluded visit #5 due to significant increase in activity and change in medications
41	1	FS	Mixed breed	Carprofen	L Hip OA	CBD-PL	Unenrolled at visit #4 due to owner withdrawal
42	2	MN	Mixed breed	Carprofen	R carpal desmopathy and tendinopathy	CBD-PL	All data excluded due to suspected inadvertent CBD administration throughout trial

CBD, cannabidiol oil; PL, placebo oil.

A summary of all subjective and objective outcome measures is reported in Tables 2, 3. Compared to baseline data, veterinary assessments (*p* = 0.044), CBPI (*p* < 0.001), CSOM (p < 0.001), and both moderate and total activity counts (*p* = 0.033 and *p* = 0.046, respectively) showed improved outcomes in dogs receiving CBD. Objective gait analysis percent body weight distribution data showed insufficient evidence of improvement for both the placebo and CBD groups (*p* = 0.197 and *p* = 0.121, respectively). The CBD group showed eight significant comparisons to baseline while the placebo group only showed three significant comparisons. The improvements seen in the placebo group compared to baseline confirm an expected caregiver placebo effect. However, there was insufficient evidence of improvement in veterinary assessments or objective outcome measures in the placebo group. Comparisons between groups found evidence of dogs receiving CBD showing improvement in veterinary assessments (*p* = 0.046), the pain severity scoring of CBPI (*p* = 0.017), and behavior scoring of CSOM (*p* = 0.007).

**Table 2:** Client metrology instruments (CMI) as means and standard error and differences between baseline, placebo and CBD for each including CBPI (PSS, PIS, QoL), CSOM (ACT, BEHAV).

CMI	Treatment	Baseline and post-treatment CMI score, Mea*n* ± SE	Post-Treatment CMI score comparison, difference ± SE	*P* value comparing between treatments
^↓^SOS Total (0–24)	Baseline	9.81 ± 0.55	Placebo-baseline 0.23 ± 0.28	0.411
	Placebo	10.04 ± 0.60	CBD – Baseline −0.55 ± 0.27	0.044 *
	CBD	9.27 ± 0.59	CBD – Placebo −0.78 ± 0.39	0.046 *
^↓^CBPI PSS (0–10)	Baseline	4.11 ± 0.23	Placebo-baseline −0.34 ± 0.23	0.143
	Placebo	3.79 ± 0.32	CBD – Baseline −1.10 ± 0.22	0.000 *
	CBD	3.05 ± 0.31	CBD – Placebo −0.77 ± 0.32	0.017 *
^↓^CBPI PIS (0–10)	Baseline	5.21 ± 0.27	Placebo – baseline −0.98 ± 0.32	0.002 *
	Placebo	4.23 ± 0.37	CBD- baseline −1.33 ± 0.31	0.000 *
	CBD	3.88 ± 0.37	CBD – Placebo −0.35 ± 0.43	0.417
^↑^ CBPI QOL (0–5)	Baseline	3.29 ± 0.11	Placebo – baseline 0.24 ± 0.13	0.061
	Placebo	3.53 ± 0.15	CBD- baseline 0.47 ± 0.12	0.000 *
	CBD	3.77 ± 0.15	CBD – Placebo 0.24 ± 0.17	0.169
^↓^CSOM ACT (1–5)	Baseline	3.01 ± 0.11	Placebo – baseline −0.36 ± 0.14	0.013 *
	Placebo	2.64 ± 0.16	CBD- baseline −0.61 ± 0.14	0.000 *
	CBD	2.39 ± 0.16	CBD – Placebo −0.25 ± 0.20	0.206
^↑^ CSOM BEHAV (1–5)	Baseline	1.99 ± 0.11	Placebo – baseline 0.46 ± 0.16	0.004 *
	Placebo	2.45 ± 0.17	CBD- baseline 1.04 ± 0.15	0.000 *
	CBD	3.03 ± 0.16	CBD – Placebo 0.58 ± 0.21	0.007 *

CBD, cannabidiol; SOS, subjective orthopedic scoring; CBPI, canine brief pain inventory; CMI, clinical metrology instrument; PSS, pain severity score; PIS, pain interference score; QOL, quality of life; CSOM, client subjective outcome measure; ACT, activity; BEHAV, behavior. Direction of arrow next to listed CMIs denotes the direction of value which indicates a more favorable response (i.e., ^↓^ represents that a lower score equates to clinical improvement and vice versa).

**Table 3:** Objective outcome measures represented by means and standard error and differences between baseline, placebo, and CBD including activity and percent body weight distribution.

Objective measure	Treatment	Baseline and post-treatment, Mea*n* ± SE	Post-treatment, difference ± SE	*P* value comparing between treatments
^↑^ OGA.BWD	Baseline	21.80 ± 0.95	Placebo-baseline 0.51 ± 0.39	0.197
	Placebo	22.31 ± 1.01	CBD – Baseline 0.61 ± 0.39	0.121
	CBD	22.41 ± 1.01	CBD – Placebo 0.10 ± 0.55	0.853
^↓^Actical.SED	Baseline	1109.75 ± 23.40	(Placebo-baseline)/Baseline −0.14% ± 1.17%	0.903
	Placebo	1108.17 ± 25.31	(CBD – Baseline)/Baseline −0.86% ± 1.07%	0.427
	CBD	1100.22 ± 24.98	(CBD – Placebo)/Baseline −0.72% ± 1.57%	0.651
^↓^Actical.Light	Baseline	171.29 ± 9.23	(Placebo-baseline)/Baseline 4.12% ± 4.02%	0.300
	Placebo	178.35 ± 10.90	(CBD – Baseline)/Baseline −3.99% ± 3.42%	0.258
	CBD	164.47 ± 9.96	(CBD – Placebo)/Baseline −7.78% ± 4.80%	0.125
^↑^ Actical.MOD	Baseline	109.53 ± 9.69	(Placebo-baseline)/Baseline −0.28% ± 6.90%	0.968
	Placebo	109.22 ± 11.20	(CBD – Baseline)/Baseline 14.98% ± 7.36%	0.033 *
	CBD	125.94 ± 12.78	(CBD – Placebo)/Baseline 15.30% ± 10.76%	0.132
^↑^ Actical.VIG	Baseline	0.48 ± 0.23	(Placebo-baseline)/Baseline 9.49% ± 12.50%	0.430
	Placebo	0.62 ± 0.29	(CBD – Baseline)/Baseline −3.26% ± 10.20%	0.754
	CBD	0.43 ± 0.25	(CBD – Placebo)/Baseline −11.65% ± 13.61%	0.424
^↑^ Total activity count	Baseline	1.17×10^5^ ± 1.36×10^4^	(Placebo-baseline)/Baseline 3.71% ± 9.58%	0.695
	Placebo	1.21×10^5^ ± 1.64×10^4^	(CBD – Baseline)/Baseline 18.98% ± 10.16%	0.046 *
	CBD	1.39×10^5^ ± 1.86×10^4^	(CBD – Placebo)/Baseline 14.73% ± 14.28%	0.274

OGA, objective gait analysis; BWD, body weight distribution; SED, sedentary; MOD, moderate; VIG, vigorous. Direction of arrow next to listed CMIs denotes the direction of value which indicates a more favorable response (i.e., ^↓^ represents that a lower score equates to clinical improvement and vice versa).

Seventeen dogs had elevations in at least one liver enzyme throughout the study (predominantly ALP), but one dog was excluded from analysis after starting corticosteroids for pemphigus foliaceus that resulted in elevated ALP following the washout period. Three patients with AST elevations, one of which also had a T-bilirubin elevation, without concurrent ALP and ALT elevations, had these single data points excluded from analysis as the sample was hemolyzed and these two markers can be affected by hemolysis (21). Characterization of the liver enzyme elevations and the associated treatment(s) are summarized in Table 4. Of the 14 patients with meaningful elevations included for analysis, 10 dogs were receiving CBD at the time of elevations, seven of which were concurrently receiving NSAIDs. Four dogs with elevations were receiving placebo and NSAID, but three of these dogs had received CBD first and continued to have elevations throughout the study, although these values were decreasing. Two dogs with elevated liver enzymes were receiving the placebo treatment alone. Both of these were ALT elevations and one dog’s elevation started after receiving CBD and having elevations in both ALP and ALT, but the ALP elevation resolved. Changes in liver enzymes for patients receiving NSAIDs, CBD, and placebo and their comparisons are summarized in Tables 5, 6. Both patients receiving CBD alone and those receiving CBD and NSAID showed evidence of ALP elevations (*p* < 0.001 for all). Additionally, there was evidence that this increase in ALP was greater in dogs receiving CBD and NSAID compared to CBD alone (*p* = 0.046). For ALT, only patients receiving CBD and NSAID showed evidence of elevation compared to NSAID administration alone (*p* = 0.022 and *p* = 0.025). Of the patients with any elevation in liver enzymes, six owners consented to focused hepatic ultrasound and five to fine needle aspirates of the liver. Changes to the liver included glycogen accumulation (*n* = 2), vacuolar hepatopathy (*n* = 5), and mild lymphocytic inflammation (*n* = 2). One dog additionally had multifocal necrosis on cytology. This patient had a mildly elevated ALP at the time of enrollment with moderate elevation following CBD administration, and fasted bile acids at the time of that elevation that were within normal limits.

**Table 4:** Case evaluation of dogs with hepatic enzyme elevations including percent ALP increase from baseline, treatment(s) at the time of elevation, hepatic ultrasound findings, hepatic cytology, and other notable changes.

Patient	% Inc ALP from baseline	Treatment(s)	Hepatic ultrasound findings	Hepatic cytology	Other notes
3	100%	CBD	Hyperechoic and coarse liver	Glycogen accumulation	Bile acids: 5 umol/L, resolved at follow up
5	N/A – ALT elevation only	Placebo	Ultrasound not performed	Not performed	Resolved at follow up
13	178%	CBD, carprofen	Mildly heterogenous hepatic parenchyma with new hyperechoic nodule	Mild–moderate hepatocellular vacuolation. Multifocal necrosis	Bile acids: 3 umol/L, ALP elevation at enrollment
14	450%	CBD, carprofen	Ultrasound not performed	Not performed	ALP 143 U/L (ref 15–140)
16	255%	CBD	Normal liver	Vacuolar hepatopathy	Bile acids 5 umol/L, ALT elevations, resolved at follow up
22	645%	CBD	Ultrasound not performed	Not performed	Resolved at follow up
23	404%	CBD, carprofen	Ultrasound not performed	Not performed	Resolved at follow up
29	N/A – ALT elevation only	Placebo, carprofen	Ultrasound not performed	Not performed	ALT 99 U/L, resolved at follow up
30	4,445%	CBD, carprofen	Mild benign change (ie vacuolar hepatopathy)	Not performed	Resolved at follow up
33	1,919%	CBD, carprofen	Non-specific, likely chronic, hepatopathy and solitary hypo- to isoechoic nodule	Moderate to marked hepatocellular vacuolization, mild lymphocytic inflammation	ALT 117 U/L
35	1,327%	CBD, carprofen	Ultrasound not performed	Not performed	
36	1,673%	CBD	Diffusely hyperechoic hepatic parenchyma with multiple hyperechoic nodules	Vacuolar hepatopathy w/ mild lymphocytic inflammation	ALT 100 U/L, all elevations resolved at follow up
40	1,392%	CBD, carprofen	Ultrasound not performed	Not performed	ALT 123 U/L

**Table 5:** Hepatic enzymes represented as means and standard error for each treatment combination measured for ALP, ALT, AST, T-bilirubin, and GGT as well as the number of dogs above the reference range for each hepatic enzyme for each treatment combination.

Hepatic Enzyme	Treatment(s)	# Dogs above reference range	Mean	SE
ALP (U/L)	Baseline & NSAID	2	43.96	9.48
	Placebo & NSAID	3	41.39	10.01
	CBD & NSAID	7	154.12	37.89
	Baseline	0	37.35	8.26
	Placebo	0	31.24	7.95
	CBD	3	80.28	19.65
ALT (U/L)	Baseline & NSAID	0	40.58	3.75
	Placebo & NSAID	3	38.60	4.05
	CBD & NSAID	2	48.80	5.22
	Baseline	0	36.91	3.50
	Placebo	2	34.91	3.87
	CBD	1	39.61	4.21
AST (U/L)	Baseline & NSAID	0	25.24	1.29
	Placebo & NSAID	0	26.02	1.60
	CBD & NSAID	0	28.36	1.79
	Baseline	0	26.04	1.36
	Placebo	0	25.96	1.70
	CBD	1	27.91	1.72
T-bilirubin (mg/dL)	Baseline & NSAID	0	0.12	0.01
	Placebo & NSAID	0	0.11	0.01
	CBD & NSAID	0	0.10	0.01
	Baseline	0	0.13	0.01
	Placebo	0	0.10	0.01
	CBD		0.09	0.01
GGT (U/L)	Baseline & NSAID	0	1.16	0.25
	Placebo & NSAID	0	0.62	0.34
	CBD & NSAID	0	0.96	0.35
	Baseline	0	0.94	0.26
	Placebo	0	0.85	0.37
	CBD	0	1.03	0.34

**Table 6:** Comparisons of hepatic enzyme changes represented as a mean percentage increase, standard error, and differences for ALP, ALT, AST, T-bilirubin, and GGT.

Hepatic Enzyme	Comparison of Differences	Mean % Increase	SE	P-Value
ALP	(Placebo & NSAID - Baseline & NSAID) / Baseline & NSAID	−5.85	16.01	0.724
	(CBD & NSAID - Baseline & NSAID) / Baseline & NSAID	250.56		0.000 *
	(CBD & NSAID - Placebo & NSAID) / Placebo & NSAID	272.34	60.93	0.000 *
	(Placebo - Baseline) / Baseline	−16.37	84.56	0.324
	(CBD - Baseline) / Baseline	114.93	15.10	0.000 *
	(CBD - Placebo) / Placebo	156.99	36.34	0.000 *
	(Baseline & NSAID - Baseline) / Baseline	17.70	59.16	0.590
	(Placebo & NSAID - Placebo) / Placebo	32.50	35.28	0.383
	(CBD & NSAID - CBD) / CBD	91.98	42.34	0.046 *
ALT	(Placebo & NSAID - Baseline & NSAID) / Baseline & NSAID	−4.87	7.38	0.521
	(CBD & NSAID - Baseline & NSAID) / Baseline & NSAID	20.27	9.53	0.022 *
	(CBD & NSAID - Placebo & NSAID) / Placebo & NSAID	26.43	13.05	0.025 *
	(Placebo - Baseline) / Baseline	−5.42	7.79	0.500
	(CBD - Baseline) / Baseline	7.32	8.28	0.362
	(CBD - Placebo) / Placebo	13.48	11.88	0.229
	(Baseline & NSAID - Baseline) / Baseline	9.93	14.08	0.464
	(Placebo & NSAID - Placebo) / Placebo	10.57	15.22	0.469
	(CBD & NSAID - CBD) / CBD	23.19	16.89	0.134
AST	(Placebo & NSAID - Baseline & NSAID) / Baseline & NSAID	3.07	5.67	0.583
	(CBD & NSAID - Baseline & NSAID) / Baseline & NSAID	12.35	6.39	0.040 *
	(CBD & NSAID - Placebo & NSAID) / Placebo & NSAID	9.00	7.79	0.230
	(Placebo - Baseline) / Baseline	−0.30	5.83	0.959
	(CBD - Baseline) / Baseline	7.20	5.85	0.206
	(CBD - Placebo) / Placebo	7.52	7.81	0.320
	(Baseline & NSAID - Baseline) / Baseline	−3.06	6.69	0.655
	(Placebo & NSAID - Placebo) / Placebo	0.22	7.84	0.977
	(CBD & NSAID - CBD) / CBD	1.60	7.83	0.838
T-bilirubin	(Placebo & NSAID - Baseline & NSAID) / Baseline & NSAID	−0.01	0.01	0.403
	(CBD & NSAID - Baseline & NSAID) / Baseline & NSAID	−0.02	0.01	0.144
	(CBD & NSAID - Placebo & NSAID) / Placebo & NSAID	−0.01	0.02	0.597
	(Placebo - Baseline) / Baseline	−0.03	0.01	0.018 *
	(CBD - Baseline) / Baseline	−0.04	0.01	0.002 *
	(CBD - Placebo) / Placebo	−0.01	0.02	0.672
	(Baseline & NSAID - Baseline) / Baseline	−0.01	0.01	0.401
	(Placebo & NSAID - Placebo) / Placebo	0.01	0.01	0.427
	(CBD & NSAID - CBD) / CBD	0.01	0.01	0.487
GGT	(Placebo & NSAID - Baseline & NSAID) / Baseline & NSAID	−0.53	0.39	0.169
	(CBD & NSAID - Baseline & NSAID) / Baseline & NSAID	−0.20	0.39	0.620
	(CBD & NSAID - Placebo & NSAID) / Placebo & NSAID	0.34	0.47	0.475
	(Placebo - Baseline) / Baseline	−0.10	0.41	0.812
	(CBD - Baseline) / Baseline	0.09	0.39	0.818
	(CBD - Placebo) / Placebo	0.19	0.48	0.699
	(Baseline & NSAID - Baseline) / Baseline	0.21	0.31	0.502
	(Placebo & NSAID - Placebo) / Placebo	−0.22	0.41	0.586
	(CBD & NSAID - CBD) / CBD	−0.07	0.41	0.858

Side effects were uncommon but included gastrointestinal signs such as vomiting and diarrhea. Two dogs were reported to vomit on CBD alone, one dog on both CBD and placebo, and one dog was reported to have diarrhea on CBD. All gastrointestinal signs appeared to be self-limiting and resolved without further intervention and while continuing to receive the product.

Batch analysis was performed on both the CBD and placebo products before being dispensed (SC Laboratories California LLC, Santa Cruz California, USA). Certificate of analysis showed no detectable levels of THC, CBD, or other cannabinoids in the placebo product (Supplementary material). Per 30 mL unit, the CBD product contained a range of 1570.62 mg total CBD and 1585.86 mg total cannabinoids. This included 8.46 mg CBG and 3.87 mg CBDV. There were no detectable levels of THC. Both the CBD and placebo products were additionally tested for the presence of pesticides, residual solvents, mycotoxins, heavy metals, foreign material, and microbiological contaminants such as bacteria, yeast, and molds. Results were passing for both products across all measures.

Thirty-nine were included for plasma analysis. One dog was excluded because the owner withdrew during the first phase of administration, one dog was excluded for returning high CBD plasma values for both placebo and CBD treatment phases, raising the concern for inadvertent CBD administration, and one dog was excluded due to low levels throughout the study which may have been due to late timing of blood draws relative to last dose or owner non-compliance. Three dogs who did not complete the study were included for plasma analysis up until the point of unenrollment. All three of these dogs received the CBD oil first, so there were 36 placebo oil samples and 39 CBD oil samples included for analysis. There was one outlier value for the placebo and two outlier values for the CBD oil samples. Outlier values were retested to confirm but ultimately included for analysis because the timing of blood draws relative to the last CBD dosing was not controlled for in this study and may have led to variation in sample values. The median CBD plasma level following the administration of CBD oil was 141.5 ng/mL (range 3.13–1850). The median for the placebo oil was below the level of quantification (BLOQ) at <0.98 ng/mL (range BLOQ-104). There was a statistically significant difference between CBD plasma levels compared to placebo levels (*p* < 0.001).

## 4 Discussion

This double-blind, crossover, placebo-controlled study was conducted to evaluate the effect of CBD in client-owned dogs with mobility disorders as well as provide more information regarding patient tolerance when co-administered with NSAIDs. For clinical relevance, this study sought to evaluate the effect of CBD on pain and function in dogs, so enrollment was expanded to all mobility impairments and not limited to just those with osteoarthritis, although the most common diagnosis in the enrolled patients. To address some of the limitations of prior studies, both subjective and objective outcome measures were used to assess dogs in a crossover design. The study results suggest a potential therapeutic benefit of CBD administration for the management of mobility impairments, as well as patient tolerance when co-administered with NSAIDs in dogs.

Several previous studies evaluating CBD for pain conditions have also found improvements in CMIs, but a recent systematic review and meta-analysis of CBD literature for canine OA found a high risk of bias in the available literature (11). Dogs in the present study with pain related to mobility impairments showed improvement in both the CBD and placebo groups. The observed improvement in the placebo group is likely attributed to an expected caregiver placebo effect which has been reported to occur up to 57% of the time when owners or veterinarians observe a dog’s lameness (22). When comparing treatment groups, however, only the CBD group showed improvement in veterinary assessments, pain severity scores, and client-specific behavior scores. Furthermore, blinded veterinary assessments showed improvements in the CBD group but not in the placebo group. The combination of these findings may indicate a positive effect of CBD on pain and function.

Objectively, this study used both a pressure sensitive walkway and accelerometry to assess dogs after administration of placebo and CBD oil. Objective gait analysis (OGA) did not show improvement in this study, but objective gait analysis is not without its limitations. While several trials were collected for each dog during each return visit, the data could theoretically be influenced by outside factors such as the dog’s activity level prior to data collection and anxiety in hospital. Kinetic data can also be influenced by factors such as walking versus trotting, the number of trials collected, handler, velocity, and acceleration (23). While the velocity for valid trials needed to be within 0.3 m/s to be considered a valid trial, acceleration was not controlled in this study.

Because gait analysis measurements occur in the hospital setting during this singular time frame at each visit, a second objective means of measuring response to CBD was selected in this study to provide more broad information regarding a dog’s activity changes at home over a longer period of time. Dogs in this study showed a significant increase in both moderate and total activity counts when receiving CBD oil compared to baseline. While accelerometry may provide more information regarding a dog’s activity over time, the output values can be influenced by factors such as erroneous reading from collar loosening, equipment malfunction, or scratching at the collar and device. It has also been suggested that the use of accelerometry as an outcome measure in clinical research is questionable as it is easily influenced by owner behaviors (increase or decrease in activity base on perceived or desired outcome) rather than a true representation of changes in pain (13). When considering changes in activity counts, however, an increase in activity by 20% was clinically relevant when accelerometry was used to measure differences in dogs with naturally occurring OA treated with carprofen versus a placebo (24). Percent increase in total activity count for the CBD group in this study approached this value (18.98% ± 10.16%) which may further support the use of CBD for the management of mobility disorders in dogs.

The dose of CBD may also contribute to the improvements seen across outcome measures in this study compared to others. Previous studies evaluating the efficacy of CBD oil for the management of pain disorders suggest a dose range of 4–5 mg/kg/day (13, 25–27). The present study used a higher dose of CBD at 10 mg/kg/day (5 mg/kg q12h). Despite higher doses, however, the observed CBD plasma concentrations in this study were similar to previously reported values, but they did show a greater range of values (26). Therapeutic plasma levels do not appear to be well established in the literature, and plasma levels may be greatly influenced by several factors such as variable absorption between patients, variations in the CBD oil product, and the timing of blood draws relative to dosing. One major limitation regarding the measurement of plasma CBD levels in this study is the timing of blood draw relative to last dosing. Owners were instructed to administer the oil the morning of the appointment, but the time of morning feeding and time of blood draw varied between patients and likely contributed to variability in plasma concentrations. While pharmacokinetics can differ between CBD products, a recent pharmacokinetic study measured CBD concentrations over a 24-h period after administration in a population of healthy laboratory beagles that revealed changes in CBD concentration over time with peak concentrations occurring around two hours after administration (28). Another study found an elimination half-life of 4.2 h at both 2 mg/kg and 8 mg/kg dosing (26). Given this information, the timing of blood draw relative to the last CBD dose likely had an impact on CBD concentrations and the variation noted. While most blood draws in this study occurred in the morning, theoretically within a few hours of CBD administration, this variable, along with timing of administration, was not controlled for in this study and should be considered in future studies. Additionally, this CBD product was considered a broad-spectrum rather than full-spectrum product and contained no reported cannabidiolic acid (CBDA) which may have influenced outcomes as CBDA is thought to be more bioavailable and may aid in the absorption of CBD (28). Product differences likely exist between the different formulations (e.g., broad-spectrum, full-spectrum, isolates) and even within different products of the same formulation. This highlights the importance of testing different products and formulations for tolerability and absorption through plasma levels. Owner compliance may have also influenced the variation of CBD plasma values. A previous study evaluating owner compliance with veterinary prescribed therapeutics found 68% of owners missed at least one dose while 14% missed a significant proportion of doses, giving less than 60% as reported by electronic monitoring. Despite having electronic monitors, these owners were also likely to self-report perfect compliance while missing at least one dose (29).

Given that NSAIDs are a common treatment for pain and may also result in liver enzyme elevations, this study sought to further evaluate patient clinical tolerance when co-administered with CBD. Previous studies have evaluated CBD safety and efficacy while allowing dogs to remain on regular NSAID therapy, but these studies did not group dogs based on their NSAID administration (13, 25, 26). Administration of CBD oil has previously been shown to result in ALP elevations in both humans and dogs, and that association was also seen in this study in both dogs on CBD and NSAID combined as well as CBD alone (26, 27, 30–34). This ALP elevation is thought to be related to the induction of cytochrome P-450 oxidative metabolism (35, 36). Interestingly, five patients in this study also had mild ALT elevations following administration of CBD. Only one of these patients had ALP elevations at the time of enrollment. Elevations in ALT were reported in a recently published article for the management of epilepsy, but this appears to be the only report in veterinary literature apart from the present study (34). As in that study, a higher dose of CBD was given here compared to the 1-2 mg/kg twice daily dosing used in most other clinical studies. While the higher doses of CBD used in this study may account for the elevations seen in ALT, a prior study evaluating high doses of CBD (10 mg/kg/day and 20 mg/kg/day) given for 6-weeks in 30 healthy beagle dogs that found no clinically significant changes in serum biochemistry parameters other than elevations in ALP (33). In this study, however, only binary statistics were performed to evaluate rises in ALP greater than a 2-fold increase from baseline. Smaller elevations in ALP, such as was evaluated in the present study, were not documented. Another recent study administered CBD at doses of 2 mg/kg and 4 mg/kg twice daily for two weeks and found no ALT elevations and ALP elevations in only 3/16 dogs receiving CBD at 4 mg/kg (37).

The present study evaluated the effect of CBD co-administered with NSAIDs and its effect on liver enzymes. Only dogs receiving CBD and NSAID together showed evidence of ALT increases. There was also evidence of greater increases in AL*P* values for patients receiving NSAIDs and CBD together compared to patients receiving CBD alone. In the study by Rozental et al., CBD was also associated with an increase in ALT when used in combination with other anti-epileptic drugs. While direct drug comparisons cannot be made between this study and the present, both phenobarbital and NSAIDs have been associated with liver enzyme elevations (38, 39). Findings from this previous study and the present may suggest interaction of CBD with other drugs to influence liver enzymes. To the authors’ knowledge, no present study exists in human or veterinary literature seeking to understand the effects of CBD and NSAID co-administration on liver enzyme elevations and its clinical relevance.

Of the patients in the present study who had liver enzyme elevations, five underwent further work-up of the liver with no apparent liver damage noted on ultrasound, cytology, or fasted bile acids testing. Three patients who returned to the teaching hospital one to six months after completion, for reasons unrelated to the study, all had normal liver enzyme values on follow up serum biochemistry. Two of these three patients were receiving NSAIDs. This would suggest the increase in ALT associated with CBD administration did not persist following cessation of the CBD. This is similar to findings in a recent safety study in which ALP elevations normalized in healthy dogs within 4 weeks of treatment cessation (30). Owners in the present study were given the option to pursue further work-up of the liver if enzyme elevations were noted, but many declined for reasons such as prolonged appointment time and possible necessity for sedation to obtain ultrasound images and/or aspirates. Another limitation of the liver work-up is the lack of long-term follow-up, and the absence of post-prandial bile acids. Therefore, limited conclusions can be drawn about the clinical significance of these liver enzyme changes with CBD and NSAIDs used together. Future studies may consider more extensive assessment of the liver to better understand the relationship between CBD use and liver enzyme elevations.

Apart from liver enzyme elevations, the only other reported adverse effect was self-limiting gastrointestinal signs. This occurred in approximately 10% of patients and required no further intervention. Of the dogs reporting GI symptoms, two were receiving NSAIDs but only reported side effects when receiving CBD. The one patient reported to vomit on both CBD and placebo oil had a history of intermittent gastrointestinal signs and was not receiving an NSAID at the time of the study. These study results suggest the co-administration of CBD and NSAIDs appears well tolerated with regard to GI side effects, but increases in liver enzymes were seen when dogs were receiving CBD and NSAID together that were greater than CBD or NSAID administration alone. Further studies are needed evaluating long term co-administration of NSAIDs and CBD before conclusions can be drawn regarding the safety of co-administration.

Given the efficacy of NSAIDs, this could be considered a confounding factor for the improvements seen across outcome measures, but the crossover design of this study sought to eliminate it as such. By enrolling dogs receiving NSAIDs consistently, as well as enrolling dogs not receiving NSAIDs, this study was able to evaluate the effects of co-administration with CBD oil. By setting the inclusion criteria of a consistent management protocol for mobility impairments and implementing a crossover design in which each patient received both CBD and placebo, we sought to eliminate the confounding factors of NSAIDs, nutraceuticals, and other pain medications.

The study results suggest a potential therapeutic benefit of CBD administration for the management of mobility impairments, however, there appeared to be an increase in ALP and ALT values in patients receiving CBD and NSAID together. While no other adverse events occurred related to the co-administration of NSAIDs and CBD, the sample size in this population is small and limits definitive conclusions. Future studies should evaluate bile acids, hepatic ultrasound, and ideally liver biopsy of patients with elevated liver enzymes following the co-administration of CBD and NSAIDs. Long term studies assessing the effect of CBD on mobility disorders in dogs are needed.

## Data Availability

The raw data supporting the conclusions of this article will be made available by the authors, without undue reservation.
