# Supplementary material for: Evaluation of the effect of cannabidiol administration with and without nonsteroidal anti-inflammatory drugs in dogs with mobility disorders: a prospective, double-blind, crossover, placebo-controlled study
Source: Front Vet Sci. 2024 Sep 25;11:1449343. doi: 10.3389/fvets.2024.1449343 (PMC11461463; doi:10.3389/fvets.2024.1449343)
Supplement: Supplementary file 1 [file Data_Sheet_1.zip › Cover Sheet.docx]

Supplementary Material

Evaluation of the effect of cannabidiol administration with and without nonsteroidal anti-inflammatory drugs in dogs with mobility disorders: A prospective, double-blind, crossover, placebo-controlled study

Bryce Talsma^1^, Lindsay Elam^1*^, Stephanie McGrath^1^ , Tianjian Zhou^2^, Craig Webb^1^, Felix Duerr^1^,

^1^Department of Clinical Sciences, Colorado State University, Fort Collins, CO, United States

^2^Department of Statistics, Colorado State University, Fort Collins, CO, United States

*** Correspondence:**Lindsay Elam
Lindsay.Elam@colostate.edu

## Supplementary Figures

**
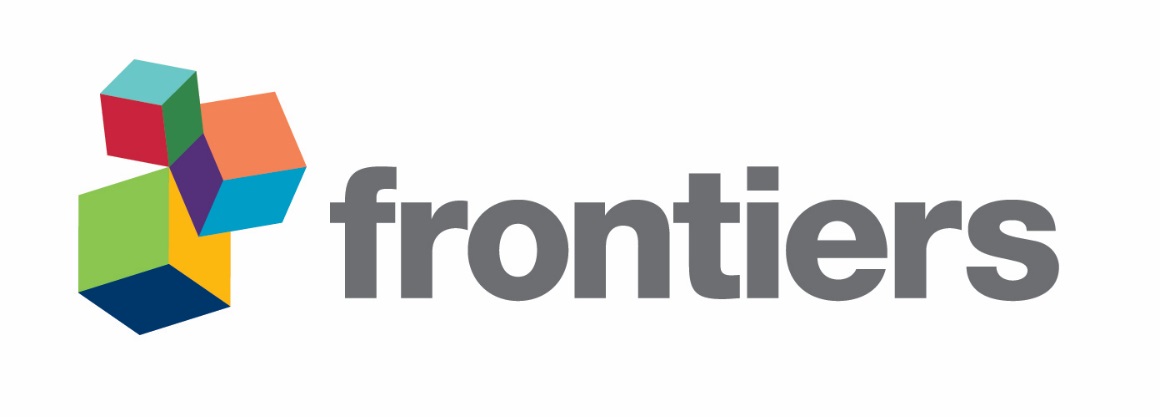
**

**Supplementary Figure 1.** Certificate of analysis for CBD product

**Supplementary Figure 2.** Certificate of analysis for placebo

**Supplementary Figure 3**: CSOM data sheet provided to owners at each visit as well as activities examples given to owners at the time of enrollment
