# Supplementary material for: Evaluation of the effect of cannabidiol administration with and without nonsteroidal anti-inflammatory drugs in dogs with mobility disorders: a prospective, double-blind, crossover, placebo-controlled study
Source: Front Vet Sci. 2024 Sep 25;11:1449343. doi: 10.3389/fvets.2024.1449343 (PMC11461463; doi:10.3389/fvets.2024.1449343)
Supplement: Supplementary file 1 [file Data_Sheet_1.zip › Supplementary Figure 2.PDF]

Figure S2. Certificate of Analysis for placebo product

Hemp Quality Assurance Testing

CERTIFICATE OF ANALYSIS

DATE ISSUED 11/08/2021

SAMPLE NAME: cbdMD Tincture Canine Peanut Butter 1500 mg Placebo
Infused, Hemp Infused

CULTIVATOR / MANUFACTURER
Business Name:
License Number:
Address:

DISTRIBUTOR / TESTED FOR
Business Name: Paw CBD
License Number:
Address:

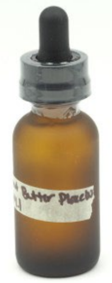

SAMPLE DETAIL

Batch Number: CBD-014.1
Sample ID: 211104M037

Date Collected: 11/04/2021
Date Received: 11/04/2021
Batch Size:
Sample Size: 1.0 units
Unit Mass:
Serving Size: 1 milliliters per Serving

Scan QR code to verify authenticity of results.

CANNABINOID ANALYSIS - SUMMARY

Total THC: **Not Detected**

Total CBD: **Not Detected**

Sum of Cannabinoids: Not Detected

Total Cannabinoids: Not Detected

Total THC/CBD is calculated using the following formulas to take into account the loss of a carboxyl group during the decarboxylation step:
Total THC = Δ9THC + (THCa (0.877))
Total CBD = CBD + (CBDa (0.877))
Sum of Cannabinoids = Δ9THC + THCa + CBD + CBDa + CBG + CBGa + THCV + THCVa + CBC + CBCa + CBDV + CBDVa + Δ8THC + CBL + CBN
Total Cannabinoids = (Δ9THC+0.877*THCa) + (CBD+0.877*CBDa) + (CBG+0.877*CBGa) + (THCV+0.877*THCVa) + (CBC+0.877*CBCa) + (CBDV+0.877*CBDVa) + Δ8THC + CBL + CBN

Density: 0.9468 g/mL

SAFETY ANALYSIS - SUMMARY

Pesticides: **PASS**
Heavy Metals: **PASS**
Foreign Material: **PASS**
Mycotoxins: **PASS**
Microbiology (PCR): **PASS**
Residual Solvents: **PASS**
Microbiology (Plating): **PASS**

For quality assurance purposes. Not a Pre-Harvest Hemp Lab Test Report. These results relate only to the sample included on this report. This report shall not be reproduced, except in full, without written approval of the laboratory.

Sample Certification: Action Limits used in this report are a compilation of guidance from state regulatory agencies in all states. Action limits for required tests are either state-specific, or the lower of any conflicting state regulations based upon the panel requested.

Decision Rule: Statements of conformity (e.g. Pass/Fail) to specifications are made in this report without taking measurement uncertainty into account. Where statements of conformity are made in this report, the following decision rules are applied: PASS –Results within limits/specifications, FAIL –Results exceed limits/specifications.

References: limit of detection (LOD), limit of quantification (LOQ), not detected (ND), not tested (NT), too numerous to count >250 cfu/plate (TNTC), colony-forming unit (cfu)

LQC verified by: Randi Vuong
Date: 11/08/2021

Approved by: Josh Wurzer, President
Date: 11/08/2021

© 2021 SC Labs all rights reserved. Trademarks referenced are trademarks of either SC Labs or their respective owners. MKT0002 REV8 6/21

Cannabinoid Analysis

Tested by high-performance liquid chromatography with diode-array detection (HPLC-DAD).

Method: QSP 1157 - Analysis of Cannabinoids by HPLC-DAD

TOTAL THC: **Not Detected**
Total THC (Δ9THC+0.877*THCa)

TOTAL CBD: **Not Detected**
Total CBD (CBD+0.877*CBDa)

TOTAL CANNABINOIDS: Not Detected
Total Cannabinoids (Total THC) + (Total CBD) + (Total CBG) + (Total THCV) + (Total CBC) + (Total CBDV) + Δ8THC + CBL + CBN

TOTAL CBG: ND
Total CBG (CBG+0.877*CBGa)

TOTAL THCV: ND
Total THCV (THCV+0.877*THCVa)

TOTAL CBC: ND
Total CBC (CBC+0.877*CBCa)

TOTAL CBDV: ND
Total CBDV (CBDV+0.877*CBDVa)

CANNABINOID TEST RESULTS - 11/06/2021

COMPOUND	LOD/LOQ (mg/mL)	MEASUREMENT UNCERTAINTY (mg/mL)	RESULT (mg/mL)	RESULT (%)
**Δ9THC**	*0.002 / 0.014*	*N/A*	**ND**	**ND**
**Δ8THC**	*0.01 / 0.02*	*N/A*	**ND**	**ND**
**THCa**	*0.001 / 0.005*	*N/A*	**ND**	**ND**
**THCV**	*0.002 / 0.012*	*N/A*	**ND**	**ND**
**THCVa**	*0.002 / 0.019*	*N/A*	**ND**	**ND**
**CBD**	*0.004 / 0.011*	*N/A*	**ND**	**ND**
**CBDa**	*0.001 / 0.026*	*N/A*	**ND**	**ND**
**CBDV**	*0.002 / 0.012*	*N/A*	**ND**	**ND**
**CBDVa**	*0.001 / 0.018*	*N/A*	**ND**	**ND**
**CBG**	*0.002 / 0.006*	*N/A*	**ND**	**ND**
**CBGa**	*0.002 / 0.007*	*N/A*	**ND**	**ND**
**CBL**	*0.003 / 0.010*	*N/A*	**ND**	**ND**
**CBN**	*0.001 / 0.007*	*N/A*	**ND**	**ND**
**CBC**	*0.003 / 0.010*	*N/A*	**ND**	**ND**
**CBCa**	*0.001 / 0.015*	*N/A*	**ND**	**ND**
**SUM OF CANNABINOIDS**			**ND**	**ND**

Serving Size: 1 milliliters per Serving

**Δ9THC per Serving**	**ND**
**Total THC per Serving**	**ND**
**CBD per Serving**	**ND**
**Total CBD per Serving**	**ND**
**Sum of Cannabinoids per Serving**	**ND**
**Total Cannabinoids per Serving**	**ND**

DENSITY TEST RESULT

0.9468 g/mL

Tested 11/06/2021

Method: QSP 7870 - Sample Preparation

© 2021 SC Labs all rights reserved. Trademarks referenced are trademarks of either SC Labs or their respective owners. MKT0002 REV8 6/21

Pesticide Analysis

Pesticide and plant growth regulator analysis utilizing high-performance liquid chromatography-mass spectrometry (HPLC-MS) or gas chromatography-mass spectrometry (GC-MS).

*GC-MS utilized where indicated.

Method: QSP 1212 - Analysis of Pesticides and Mycotoxins by LC-MS or QSP 1213 - Analysis of Pesticides by GC-MS

Exclusions[1] see last page

Exclusions[2] see last page

PESTICIDE TEST RESULTS - 11/06/2021 PASS

COMPOUND	LOD/LOQ (µg/g)	ACTION LIMIT (µg/g)	MEASUREMENT UNCERTAINTY (µg/g)	RESULT (µg/g)	RESULT
Abamectin	0.03 / 0.10	0.3	N/A	ND	PASS
Acephate	0.02 / 0.07	5	N/A	ND	PASS
Acequinocyl	0.02 / 0.07	4	N/A	ND	PASS
Acetamiprid	0.02 / 0.05	5	N/A	ND	PASS
Aldicarb	0.03 / 0.08	≥ LOD	N/A	ND	PASS
Azoxystrobin	0.02 / 0.07	40	N/A	ND	PASS
Bifenazate	0.01 / 0.04	5	N/A	ND	PASS
Bifenthrin	0.02 / 0.05	0.5	N/A	ND	PASS
Boscalid	0.03 / 0.09	10	N/A	ND	PASS
Captan	0.19 / 0.57	5	N/A	ND	PASS
Carbaryl	0.02 / 0.06	0.5	N/A	ND	PASS
Carbofuran	0.02 / 0.05	≥ LOD	N/A	ND	PASS
Chlorantraniliprole	0.04 / 0.12	40	N/A	ND	PASS
Chlordane*	0.03 / 0.08	≥ LOD	N/A	ND	PASS
Chlorfenapyr*	0.03 / 0.10	≥ LOD	N/A	ND	PASS
Chlorpyrifos	0.02 / 0.06	≥ LOD	N/A	ND	PASS
Clofentezine	0.03 / 0.09	0.5	N/A	ND	PASS
Coumaphos	0.02 / 0.07	≥ LOD	N/A	ND	PASS
Cyfluthrin	0.12 / 0.38	1	N/A	ND	PASS
Cypermethrin	0.11 / 0.32	1	N/A	ND	PASS
Daminozide	0.02 / 0.07	≥ LOD	N/A	ND	PASS
DDVP (Dichlorvos)	0.03 / 0.09	≥ LOD	N/A	ND	PASS
Diazinon	0.02 / 0.05	0.2	N/A	ND	PASS
Dimethoate	0.03 / 0.08	≥ LOD	N/A	ND	PASS
Dimethomorph	0.03 / 0.09	20	N/A	ND	PASS
Ethoprop(hos)	0.03 / 0.10	≥ LOD	N/A	ND	PASS
Etofenprox	0.02 / 0.06	≥ LOD	N/A	ND	PASS
Etoxazole	0.02 / 0.06	1.5	N/A	ND	PASS
Fenhexamid	0.03 / 0.09	10	N/A	ND	PASS
Fenoxycarb	0.03 / 0.08	≥ LOD	N/A	ND	PASS
Fenpyroximate	0.02 / 0.06	2	N/A	ND	PASS
Fipronil	0.03 / 0.08	≥ LOD	N/A	ND	PASS
Flonicamid	0.03 / 0.10	2	N/A	ND	PASS
Fludioxonil	0.03 / 0.10	30	N/A	ND	PASS
Hexythiazox	0.02 / 0.07	2	N/A	ND	PASS
Imazalil	0.02 / 0.06	≥ LOD	N/A	ND	PASS
Imidacloprid	0.04 / 0.11	3	N/A	ND	PASS
Kresoxim-methyl	0.02 / 0.07	1	N/A	ND	PASS
Malathion	0.03 / 0.09	5	N/A	ND	PASS
Metalaxyl	0.02 / 0.07	15	N/A	ND	PASS
Methiocarb	0.02 / 0.07	≥ LOD	N/A	ND	PASS

Continued on next page

© 2021 SC Labs all rights reserved. Trademarks referenced are trademarks of either SC Labs or their respective owners. MKT0002 REV8 6/21

Pesticide Analysis *Continued*

Pesticide and plant growth regulator analysis utilizing high-performance liquid chromatography-mass spectrometry (HPLC-MS) or gas chromatography-mass spectrometry (GC-MS).

*GC-MS utilized where indicated.

Method: QSP 1212 - Analysis of Pesticides and Mycotoxins by LC-MS or QSP 1213 - Analysis of Pesticides by GC-MS

PESTICIDE TEST RESULTS - 11/06/2021 *continued* **PASS**

COMPOUND	LOD/LOQ (μg/g)	ACTION LIMIT (μg/g)	MEASUREMENT UNCERTAINTY (μg/g)	RESULT (μg/g)	RESULT
Methomyl	0.03 / 0.10	0.1	N/A	ND	PASS
Methyl parathion	0.03 / 0.10	≥ LOD	N/A	ND	PASS
Mevinphos	0.03 / 0.09	≥ LOD	N/A	ND	PASS
Myclobutanil	0.03 / 0.09	9	N/A	ND	PASS
Naled	0.02 / 0.07	0.5	N/A	ND	PASS
Oxamyl	0.04 / 0.11	0.2	N/A	ND	PASS
Paclobutrazol	0.02 / 0.05	≥ LOD	N/A	ND	PASS
Pentachloronitrobenzene*	0.03 / 0.09	0.2	N/A	ND	PASS
Permethrin	0.04 / 0.12	20	N/A	ND	PASS
Phosmet	0.03 / 0.10	0.2	N/A	ND	PASS
Piperonylbutoxide	0.02 / 0.07	8	N/A	ND	PASS
Prallethrin	0.03 / 0.08	0.4	N/A	ND	PASS
Propiconazole	0.02 / 0.07	20	N/A	ND	PASS
Propoxur	0.03 / 0.09	≥ LOD	N/A	ND	PASS
Pyrethrins	0.04 / 0.12	1	N/A	ND	PASS
Pyridaben	0.02 / 0.07	3	N/A	ND	PASS
Spinetoram	0.02 / 0.07	3	N/A	ND	PASS
Spinosad	0.02 / 0.07	3	N/A	ND	PASS
Spiromesifen	0.02 / 0.05	12	N/A	ND	PASS
Spirotetramat	0.02 / 0.06	13	N/A	ND	PASS
Spiroxamine	0.03 / 0.08	≥ LOD	N/A	ND	PASS
Tebuconazole	0.02 / 0.07	2	N/A	ND	PASS
Thiacloprid	0.03 / 0.10	≥ LOD	N/A	ND	PASS
Thiamethoxam	0.03 / 0.10	4.5	N/A	ND	PASS
Trifloxystrobin	0.03 / 0.08	30	N/A	ND	PASS

Mycotoxin Analysis

Mycotoxin analysis utilizing high-performance liquid chromatography-mass spectrometry (HPLC-MS).

Method: QSP 1212 - Analysis of Pesticides and Mycotoxins by LC-MS

Exclusions[3] see last page

MYCOTOXIN TEST RESULTS - 11/06/2021 **PASS**

COMPOUND	LOD/LOQ (μg/kg)	ACTION LIMIT (μg/kg)	MEASUREMENT UNCERTAINTY (μg/kg)	RESULT (μg/kg)	RESULT
Aflatoxin B1	2.0 / 6.0		N/A	ND	
Aflatoxin B2	1.8 / 5.6		N/A	ND	
Aflatoxin G1	1.0 / 3.1		N/A	ND	
Aflatoxin G2	1.2 / 3.5		N/A	ND	
Total Aflatoxin		20		ND	PASS
Ochratoxin A	6.3 / 19.2	20	N/A	ND	PASS

© 2021 SC Labs all rights reserved. Trademarks referenced are trademarks of either SC Labs or their respective owners. MKT0002 REV8 6/21

Residual Solvents Analysis

Residual Solvent analysis utilizing gas chromatography-mass spectrometry (GC-MS).

Method: QSP 1204 - Analysis of Residual Solvents by GC-MS

Exclusions[4] see last page

RESIDUAL SOLVENTS TEST RESULTS - 11/06/2021 PASS

COMPOUND	LOD/LOQ (µg/g)	ACTION LIMIT (µg/g)	MEASUREMENT UNCERTAINTY (µg/g)	RESULT (µg/g)	RESULT
Propane	10 / 20	5000	N/A	ND	PASS
Butane	10 / 50	5000	N/A	ND	PASS
Pentane	20 / 50	5000	N/A	ND	PASS
Hexane	2 / 5	290	N/A	ND	PASS
Heptane	20 / 60	5000	N/A	ND	PASS
Benzene	0.03 / 0.09	1	N/A	ND	PASS
Toluene	7 / 21	890	N/A	ND	PASS
Total Xylenes	50 / 160	2170	N/A	ND	PASS
Methanol	50 / 200	3000	N/A	ND	PASS
Ethanol	20 / 50	5000	± 26.1	687	PASS
Isopropyl Alcohol	10 / 40	5000	N/A	ND	PASS
Acetone	20 / 50	5000	N/A	ND	PASS
Ethyl ether	20 / 50	5000	N/A	ND	PASS
Ethylene Oxide	0.3 / 0.8	1	N/A	ND	PASS
Ethyl acetate	20 / 60	5000	N/A	ND	PASS
Chloroform	0.1 / 0.2	1	N/A	ND	PASS
Methylene chloride	0.3 / 0.9	1	N/A	ND	PASS
Trichloroethylene	0.1 / 0.3	1	N/A	ND	PASS
1,2-Dichloroethane	0.05 / 0.1	1	N/A	ND	PASS
Acetonitrile	2 / 7	410	N/A	ND	PASS

Heavy Metals Analysis

Heavy metal analysis utilizing inductively coupled plasma-mass spectrometry (ICP-MS).

Method: QSP 1160 - Analysis of Heavy Metals by ICP-MS

HEAVY METALS TEST RESULTS - 11/05/2021 PASS

COMPOUND	LOD/LOQ (µg/g)	ACTION LIMIT (µg/g)	MEASUREMENT UNCERTAINTY (µg/g)	RESULT (µg/g)	RESULT
Arsenic	0.02 / 0.1	0.42	N/A	ND	PASS
Cadmium	0.02 / 0.05	0.27	N/A	ND	PASS
Lead	0.04 / 0.1	0.5	N/A	ND	PASS
Mercury	0.002 / 0.01	0.4	N/A	ND	PASS

Microbiology Analysis

PCR AND PLATING

Analysis conducted by polymerase chain reaction (PCR) and fluorescence detection of microbiological contaminants.

Method: QSP 1221 - Analysis of Microbiological Contaminants

MICROBIOLOGY TEST RESULTS (PCR) - 11/08/2021 PASS

COMPOUND	ACTION LIMIT	RESULT	RESULT
Shiga toxin-producing *Escherichia coli*	Not Detected in 1g	ND	PASS
Salmonella spp.	Not Detected in 1g	ND	PASS
Listeria monocytogenes	Not Detected in 1g	ND	PASS

© 2021 SC Labs all rights reserved. Trademarks referenced are trademarks of either SC Labs or their respective owners. MKT0002 REV8 6/21

Microbiology Analysis *Continued*

PCR AND PLATING

Analysis conducted by 3M™ Petrifilm™ and plate counts of microbiological contaminants.

Method: QSP 6794 - Plating with 3M™ Petrifilm™

MICROBIOLOGY TEST RESULTS (PLATING) - 11/08/2021 PASS

COMPOUND	ACTION LIMIT (cfu/g)	RESULT (cfu/g)	RESULT
Total Aerobic Bacteria	*100*	ND	PASS
Total Yeast and Mold	*10*	ND	PASS

Foreign Material Analysis

Visual analysis includes, but is not limited to, sand, soil, cinders, dirt, mold, hair, insect fragments, and mammalian excreta.

Method: QSP 1226 - Analysis of Foreign Material in Cannabis and Cannabis Products

FOREIGN MATERIAL TEST RESULTS - 11/05/2021 PASS

COMPOUND	ACTION LIMIT	RESULT
Total Sample Area Covered by Sand, Soil, Cinders, or Dirt	*>25%*	PASS
Total Sample Area Covered by Mold	*>25%*	PASS
Total Sample Area Covered by an Imbedded Foreign Material	*>25%*	PASS
Insect Fragment Count	*> 1 per 3 grams*	PASS
Hair Count	*> 1 per 3 grams*	PASS
Mammalian Excreta Count	*> 1 per 3 grams*	PASS

NOTES

1. Exclusions: QSP 1213 - Sample Certification: California Code of Regulation Title 4 Division 19
2. Exclusions: QSP 1212 - Sample Certification: California Code of Regulation Title 4 Division 19
3. Exclusions: Sample Certification: California Code of Regulation Title 4 Division 19
4. Exclusions: Sample Certification: California Code of Regulation Title 4 Division 19

© 2021 SC Labs all rights reserved. Trademarks referenced are trademarks of either SC Labs or their respective owners. MKT0002 REV8 6/21
