# Supplementary material for: Evaluation of the effect of cannabidiol administration with and without nonsteroidal anti-inflammatory drugs in dogs with mobility disorders: a prospective, double-blind, crossover, placebo-controlled study
Source: Front Vet Sci. 2024 Sep 25;11:1449343. doi: 10.3389/fvets.2024.1449343 (PMC11461463; doi:10.3389/fvets.2024.1449343)
Supplement: Supplementary file 1 [file Data_Sheet_1.zip › Supplementary Figure 3.PDF]

Figure S3. CSOM data sheet provided to owners at each visit as well as activities examples given to owners at the time of enrollment

Client-Specific Outcome Measures (CSOM)

Patient Sticker

Please describe 5 time- and place-specific activities that have become altered or problematic for your dog since he/she has begun developing osteoarthritis. For example, in- stead of describing stair-climbing ability as ''stair-climbing,'' the time and place that this was noticed would include: ''ability to climb up the steps at the back of the deck in the evening.'' Also please indicate at what age you would consider your dog's activity to be completely "normal."

Comparison to when he/she was ____ years old.

1 = No problem, 2 = A little problematic, 3 = Quite problematic, 4 = Severely problematic, 5 = Impossible

Problems in mobility	*Visit 1* DATE: ________	*Visit 2* DATE: ________	*Visit 3* DATE: ________	*Visit 4* DATE: ________	*Visit 5* DATE: ________
1.					
2.					
3.					
4.					
5.					

Client-Specific Outcome Measures (CSOM)

Patient Sticker

In a manner similar to the activity survey above, please describe 3 time- and place-specific behaviors that have changed since your dog began developing osteoarthritis. These can be old behaviors that have changed or new behaviors that have developed since the onset of disease. Also please indicate at what age you would consider your dog's behavior to be completely "normal."

Comparison to when he/she was ____ years old.

1 = Significantly less than normal, 2 = Less than normal, 3 = Normal amount, 4 = More than normal, 5 = Significantly more than normal

Changed behavior as a result of osteoarthritis	*Visit 1*<br>DATE:<br>__________	*Visit 2*<br>DATE:<br>__________	*Visit 3*<br>DATE:<br>__________	*Visit 4*<br>DATE:<br>__________	*Visit 5*<br>DATE:<br>__________
1.					
2.					
3.					

Pick 5 options from this list for your CSOM

Note: If you have an activity that is relevant to your dog and not on this list, please discuss it with Clinical Trials to confirm that it will work.

Activity:

- Ability to climb the stairs
- Ability to descend the stairs
- Ability to jump on the bed (or couch)
- Ability to jump off the bed (or couch)
- Ability to jump in vehicle
- Ability to jump out of vehicle
- Ability to walk ____ miles
- Ability to posture for bowel movements
- Ability to rise without help
- Ability to keep up during walks
- Ability to stand for a long period of time
- Ability to walk without bunny hopping
- Ability to put weight on problematic limb (specify limb)
- Ability to play with other dogs

Behavior:

- Not willing to play with other dogs
- Reactive when hip/elbows/affected joint is touched
- Not eager to greet at the door/go out for walks
